# A “Hot Spring” Mimetic Aerogel Loaded with Multiple Amino Acids Modulates the Chronic Inflammatory Microenvironment Through Metabolic Reprogramming

**DOI:** 10.1002/advs.202522481

**Published:** 2026-02-03

**Authors:** Bo Li, Dandan Jin, Fei Li, Jianliang Shen, Jiang Chang, Chen Yang, Shixuan Chen, Yi Zhang

**Affiliations:** ^1^ Department of Burn and Plastic Surgery Department of Wound Repair Surgery Affiliated Hospital of Nantong University Nantong China; ^2^ Zhejiang Engineering Research Center for Tissue Repair Materials Wenzhou Institute University of the Chinese Academy of Sciences Wenzhou Zhejiang China; ^3^ Department of Gastroenterology Affiliated Hospital and Medical School of Nantong University Nantong Jiangsu China; ^4^ National Engineering Research Center of Ophthalmology and Optometry Eye Hospital, Wenzhou Medical University Wenzhou Zhejiang China

**Keywords:** amino acid, bioceramic, cell metabolism, chronic wound, photothermal therapy

## Abstract

Chronic wounds, such as diabetic foot ulcers, often exhibit lower temperatures due to impaired local circulation, leading to reduced cellular metabolism and delayed healing. This study explores a new strategy to accelerate wound healing by enhancing the cell metabolism of essential amino acids through a “hot spring” mimetic photothermal effect. We engineered an extracellular matrix mimetic nanofiber aerogel co‐delivering an eight essential‐amino‐acid cocktail and photothermally active SrCuSi4O_10_ bioceramic particles to support chronic wound repair. In vitro, fibroblasts cultured at 25°C versus 37°C showed that physiological temperature preserved the pro‐healing bioactivity of the amino‐acid treatment with minimal cytotoxicity and was associated with enhanced glutathione redox metabolism. In vivo, the amino acid–loaded aerogel combined with SrCuSi_4_O_10_‐mediated photothermal therapy accelerated wound closure and improved regenerative outcomes, including increased cellular proliferation, collagen deposition, and neovascularization. Immunohistochemical analysis showed a reduction in neutrophil infiltration, M1 macrophage polarization, and pro‐inflammatory cytokines, while increasing M2 macrophage polarization and anti‐inflammatory cytokine production, thus shifting the local inflammatory response from pro‐inflammatory to regenerative. This approach demonstrates potential as an effective therapeutic option for enhancing the healing of chronic wounds.

## Graphical abstract

The SrCuSi_4_O₁₀ bioceramic particles mimic the effects of hot spring therapy by increasing the local wound temperature, which enhances the absorption of amino acids and stimulates cell metabolism. The amino acid uptake supports cellular glutathione redox reactions, helping to mitigate oxidative stress. Additionally, this process shifts the local inflammatory response from a pro‐inflammatory to a pro‐regenerative state, creating an optimal wound bed for the repair of diabetic chronic wounds.

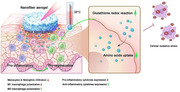

## 1 Introduction

Skin wound repair is a dynamic and complex process, typically divided into three phases: inflammation, proliferation, and remodeling. Inflammation is crucial in wound healing, initiating a series of immune responses that promote tissue repair. This phase begins immediately after injury and plays multiple essential roles in the healing process. However, unlike acute inflammation, chronic inflammation is a prolonged and dysregulated immune response [1, 2]. In chronic wounds, immune cells like macrophages and T cells often exhibit abnormal behavior, such as excessive secretion of pro‐inflammatory mediators or impaired removal of dead cells. Additionally, chronic inflammation leads to continuous activation of immune cells, creating a self‐sustaining cycle that damages tissue and disrupts normal healing [3]. Over the past decade, numerous strategies have been explored to modulate chronic inflammation in wounds. Exogenous bioactive factors are commonly used to intervene directly in the inflammatory response. For instance, natural bioactive compounds with anti‐inflammatory and antioxidant properties modulate the chronic inflammatory microenvironment [4, 5]. However, these treatments often address only the symptoms rather than the underlying cause, leading to temporary improvements and a risk of recurrence.

Wound healing is also a metabolically demanding process [6], with cell metabolism playing a key role in mitochondrial function, energy production, and the activation of pathways necessary for tissue repair [7, 8]. During skin wound healing, cells undergo metabolic reprogramming, utilizing nutrients like glucose, lipids, and amino acids to support the proliferation, differentiation, and migration of various cell types [9, 10]. Amino acids, in particular, are essential for collagen synthesis, extracellular matrix (ECM) production, wound healing, and tissue remodeling [11]. For example, alanine and glutamine can be catabolized into intermediates that fuel the tricarboxylic acid (TCA) cycle, enhancing ATP production and providing energy for cellular activities [12]. Arginine serves as a precursor for nitric oxide, a molecule crucial for vascular function and cellular signaling [13]. while cysteine contributes to glutathione synthesis, a vital antioxidant that maintains cellular redox balance. By regulating oxidative stress, glutathione protects cells from damage and supports optimal metabolic function [14]. Therefore, metabolic reprogramming is expected to improve the inflammatory microenvironment for diabetic skin wound repair, providing a foundation for effective tissue regeneration and healing.

In clinical settings, patients with chronic wounds, such as diabetic foot ulcers, often experience lower wound temperatures due to poor local blood circulation [15, 16]. Studies have shown that increasing the local temperature of chronic wounds can accelerate healing [17, 18]. Metabolic reprogramming of amino acids during skin wound healing critically influences key repair processes, such as re‐epithelialization, collagen deposition, and angiogenesis [11]. We speculate that the low wound temperatures hinder nutrient uptake by cells and slow down cellular metabolism, thereby impairing wound healing. Based on this, we hypothesis that enhancing wound repair by exogenously adding a variety of amino acids and simultaneously increasing the local wound temperature using photothermal therapy. This approach aims to improve amino acid uptake and metabolism, thus creating a more favorable microenvironment for wound healing. We discovered that short‐fiber aerogels fabricated via electrospinning successfully combine the nanoscale fiber architecture with a 3D porous network. Electrospinning allows precise control over fiber diameter, alignment, and composition, enabling the fabrication of scaffolds that closely recapitulate key structural and functional features of the native extracellular matrix. Compared with conventional hydrogels or sponges, electrospun aerogels provide a substantially higher surface‐area‐to‐volume ratio, improved mechanical integrity, and enhanced permeability to oxygen and nutrients. Moreover, their interconnected porous network efficiently absorbs wound exudate and helps maintain a moist microenvironment, which is essential for granulation tissue formation and re‐epithelialization [19]. In recent reports, SrCuSi_4_O_10_ bioceramic particles have been shown to exhibit stable and efficient photothermal conversion under near‐infrared irradiation, while also demonstrating favorable biocompatibility and lower cytotoxicity [20]. In this study, we selected eight essential amino acids (Table S1) and synthesized bioceramic particles with photothermal properties. These components were incorporated into extracellular matrix‐mimicking short fiber aerogels to investigate their effects on diabetic wound healing. Additionally, to understand how temperature influences amino acid metabolism, we cultured primary fibroblasts at 25°C and 37°C, using metabolomics to identify potential pro‐healing mechanisms.

## 2 Experimental Section

### 2.1 Materials and Reagents

Trifluoroethanol (catalog no. T109510) was obtained from Aladdin (Shanghai, China), and polycaprolactone (PCL, MW: 80 kDa) was purchased from Sigma‐Aldrich (St. Louis, USA). GelMA (catalog no. DS100) was sourced from Suhe Biotechnology (Zhejiang, China), while glacial acetic acid (catalog no. A80295) was acquired from Macklin (Shanghai, China). Glutaraldehyde, 2959, and penicillin‐streptomycin (catalog no. G4003) were purchased from Servicebio (Wuhan, China). Fetal bovine serum (FBS, catalog no. 10099–141C) and Eagle's minimum essential medium (catalog no. 11965118) were obtained from Gibco (Shanghai, China). Masson's trichrome staining solution (catalog no. MST‐8003) was provided by MXB (Fuzhou, China). Anti‐CD45 rabbit pAb (catalog no. ab10558), anti‐Ki67 rabbit pAb (catalog no. ab16667), and anti‐CCR7 rabbit pAb (catalog no. ab253187) were purchased from Abcam (Cambridge, UK). Anti‐K6 rabbit monoclonal antibody (catalog no. bsm‐60235R) was sourced from Beijing Bioss, and anti‐CD206 rabbit monoclonal antibody (catalog no. PA5‐101657) was obtained from Thermo Fisher Scientific (California, USA). Anti‐IL‐4 rabbit monoclonal antibody (catalog no. TA5142M), anti‐IL‐6 rabbit antibody (catalog no. TD6084), anti‐IL‐10 rabbit pAb (catalog no. TD6894), and anti‐TNF‐α rabbit pAb (catalog no. TA7014) were purchased from Abmart (Shanghai, China), while anti‐Ly6G rabbit pAb (catalog no. 551459) was acquired from BD Pharmingen (California, USA).

### 2.2 Synthesis of SrCuSi_4_O_10_ Particles

Mix 10 mL of SrCl_2_·6H_2_O solution (0.5 mol/L), 40 mL of Na_2_SiO_3_·9H_2_O solution (0.5 mol/L), and 0.4 g of CuO powder thoroughly by stirring. Adjust the pH of the mixture to 7 using HCl (0.5 mol/L), and continue stirring for 20 min. Then, use NH_4_OH to adjust the final pH of the solution to 12. Transfer the mixture to a reaction vessel and heat it at 250°C for 48 h. After cooling to room temperature, centrifuge at 1000 rpm for 5 min. Wash the particles with deionized water three times. After freeze‐drying, a light blue powder of SrCuSi_4_O_10_ is obtained.

### 2.3 Characterization of SrCuSi_4_O_10_ Particles

A scanning electron microscope (SEM) is used to observe the surface morphology of the particles. The particles are attached to conductive adhesive, which is then placed on the sample stage. Platinum is used as the target material, and after vacuum sputtering for 120 s, the particles are observed using the SEM (SU8010, HITACHI, Japan). A nanoparticle size analyzer (Zetasizer Nano ZS ZEN3600, Malvern, England) is used to measure the particle size and distribution of the prepared particles. The particles are dispersed in a water solution, with the concentration adjusted to 1 ppm, and the particle size distribution is then measured. The absorption spectrum of the prepared particles is measured using a UV–vis spectrophotometer. First, the particles are dispersed in a water solution at a concentration of 1 mg/mL, with water used as the background sample. The measurement is performed over a wavelength range of 200–2000 nm. An X‐ray diffractometer (XRD, D8 ADVANCE, Bruker, Germany) is used to obtain information on the structural composition of the sample particles. The samples are dried in an oven at 60°C, then ground into fine powder using a mortar. A 100 mg sample is placed on a glass slide and pressed tightly with a cover slip. The XRD analysis is performed with monochromatic Cu‐Kα radiation at 40 kV and 40 mA, with a scanning speed of 0.5°/min over a 2θ range of 10°‐80°. A Fourier‐transform infrared spectrometer (FTIR, Tensor II, Bruker, Germany) is used to measure the functional groups of the prepared particles. The samples are dried in an oven at 60°C and then ground into fine powder using a mortar. A 50 mg powder sample is placed under a diamond probe, compressed with the probe, and measured over a wavelength range of 400–4000 nm.

### 2.4 Aerogel Preparation

To fabricate the aerogel, GelMA and PCL were mixed at a 1:1 mass ratio and dissolved in trifluoroethanol to form a spinning solution. This solution was then electrospun into PCL‐GelMA nanofiber membranes, which were frozen in liquid nitrogen and cryosectioned into short fibers approximately 35 µm in width. The short fibers were dispersed in distilled water and processed using a cell disruptor to create a short‐fiber suspension. For gel formation, the suspension supplemented with 0.5% (w/v) Irgacure 2959 was transferred into a porous mold with a diameter of 8 mm, crosslinked under UV light, and freeze‐dried to produce short‐fiber aerogels. To prepare the amino acid–loaded aerogel (Aerogel + AA), 150 µL of electrospun short‐fiber suspension was used as the structural matrix, into which 50 µL of a saturated solution containing eight essential amino acids was incorporated, followed by the same processing steps as described above. Furthermore, by introducing 20 µg of SrCuSi_4_O_10_ particles into each sample, we successfully developed a dual‐functional aerogel (Aerogel + AA + BC) containing both amino acids and SrCuSi_4_O_10_ particles.

### 2.5 SEM Characterization of Aerogels

The morphology of the aerogel samples was analyzed using scanning electron microscopy (SEM). Prior to imaging, the samples were coated with a platinum layer for 1 min using a high‐vacuum ion sputter coater (Leica EM ACE600). SEM imaging was performed with a Phenom Pharos desktop scanning electron microscope, operating at an accelerating voltage of 5 kV. The resulting SEM images were then examined to assess the physical structure and elemental composition of the aerogels.

### 2.6 Liquid Absorption Capacity

Liquid absorption capacity. Freeze‐dried aerogel discs (Aerogel+AA and Aerogel+AA+BC) were prepared with identical dimensions and equilibrated to constant mass in a desiccator. Each sample was weighed to obtain the initial dry mass (*W*_0_) and then fully immersed in phosphate‐buffered saline (PBS, pH 7.4) at room temperature. At predetermined time points (5, 10, 30, and 60 min), samples were removed, gently blotted with filter paper to eliminate surface‐adhered PBS without compressing the porous structure, and immediately weighed to record the wet mass (*W*_t_). The liquid absorption (%) was calculated as:

$$\mathrm{Water}\;\mathrm{absorption}(\%)\;=\frac{W_{t}-W_{0}}{W_{0}}\times 100\%$$

Three independent samples were tested per group (*n* = 3), and results are reported as mean values.

### 2.7 Uniaxial Compression Testing of Hydrated Aerogel

Cylindrical aerogel discs (Ø 8 mm; thickness ≈2 mm) of Aerogel+AA and Aerogel+AA+BC were prepared as described and equilibrated in PBS (pH 7.4) for 24 h. After gently blotting, samples were tested in uniaxial compression at 25°C on a universal testing machine (100 N load cell). The cross‐head speed was 1 mm min^−^^1^ up to 80% engineering strain. Compressive stress was calculated as force divided by the initial cross‐sectional area; the tangent modulus (compressive modulus) was taken from the 10–20% strain region. At least three independent specimens per group were measured (n ≥ 3).

### 2.8 Release Kinetics Study

The release behavior of amino acids and particles from the aerogels was investigated under different temperature conditions. Briefly, equal‐weight aerogel samples (*n* = 3) were immersed in 10 mL of phosphate‐buffered saline (PBS, pH 7.4) and incubated at either 25°C or 37°C. At 2, 4, 8, and 12 h, 1 mL of the release medium was withdrawn and replaced with an equal volume of fresh PBS to maintain sink conditions. The concentrations of amino acids in the supernatant were quantified using a UV–vis spectrophotometer at the corresponding absorption wavelengths based on standard calibration curves. The cumulative release profile was then calculated and plotted as a function of time.

### 2.9 In Vitro and In Vivo Photothermal Effects

The dual‐loaded strontium copper silicate and amino acid aerogel (8 mm in diameter) was secured onto a low‐temperature foam board to maintain stability during laser irradiation. Photothermal response testing was conducted using a Ningbo Yuanming 1064 nm laser with power settings of 100, 200, 300, 400, 500, and 600 mW, each applied for 1 min. The surface temperature was monitored in real‐time with a FLIR A300 thermal imaging camera, and temperature data from the first 30 s were analyzed using GraphPad Prism to generate temperature‐time curves for comparison across power settings. To assess the material's photothermal response in diabetic mouse wounds, the aerogel was applied to wounds on the dorsal side of diabetic mice. The same laser was used with power settings of 100, 200, 300, and 400 mW, applied for 1 min each. A FLIR A300 thermal imaging camera recorded the maximum and minimum wound temperatures, and temperature‐time curves were generated. To prevent low‐temperature burns, a power setting that maintained a wound temperature of approximately 39°C was selected for further experiments, with each wound irradiated for 10 min daily to ensure consistency and validity in the experimental outcomes.

### 2.10 Isolation and Culture of Primary Fibroblasts from SD Neonatal Rats

First, SD neonatal rats were immersed in 75% ethanol for 5 min for disinfection, then transferred to a biosafety cabinet, where they were positioned dorsal side up with their limbs secured. The dorsal skin was excised using scissors, and excess subcutaneous tissue was removed. The skin was cut into strips approximately 3 mm wide and placed epidermis‐side up in a culture dish. A 0.25% trypsin solution was added, and the tissue was digested overnight at 4°C. After 24 h, the epidermis and dermis were manually separated, with the epidermis appearing silvery and the dermis translucent. Both layers were washed 2–3 times with PBS buffer, and the dermal tissue was minced. The minced dermal tissue was then digested in a 0.2% collagenase solution at 37°C for 30 min. The digested tissue was pipetted several times to further break it down, followed by filtration through a 200‐mesh sieve to remove any remaining debris. The filtrate was centrifuged at 1000 rpm for 5 min, and the dermal cells were collected. The supernatant was discarded, and the cells were resuspended in DMEM supplemented with 20% fetal bovine serum (FBS). The digested dermal tissue was evenly spread in 6 cm culture dishes with 2 mL of complete medium and incubated at 37°C in a 5% CO_2_ incubator for 24 h. On the second day, the medium was replenished to 5 mL, and culture was continued. After 3–4 days, fibroblasts began migrating from the tissue. On day 7, the tissue fragments were removed, and the fibroblasts were cultured with medium changes every 2–3 days until the cells reached 80%‐90% confluence.

Fibroblasts were divided into three groups: control (blank medium), medium containing 25°C aerogel extract, and medium containing 37°C aerogel extract. For the scratch assay, confluent fibroblast monolayers in 6‐well plates were scratched with a sterile pipette tip, washed, and then cultured in the corresponding media; wound closure was imaged at 0 h and 24 h and quantified using ImageJ. For the live/dead assay, fibroblasts seeded in 24‐well plates were incubated with the same three media for 24 h, stained with Calcein‐AM (live, green) and PI (dead, red), and observed under a fluorescence microscope to assess cell viability.

Following passaging, we obtained 12 T75 flasks of second‐generation fibroblasts. A pre‐prepared amino acid mixture was added to each flask, ensuring a final concentration of 10 mm for each amino acid in the medium. The flasks were divided into two groups: a 25°C group and a 37°C group, with 6 flasks in each group. The flasks were incubated in a 25°C or 37°C incubator for 24 h. After incubation, the medium was quickly aspirated, and the cells were washed 3 times with pre‐chilled PBS at 4°C. Residual PBS was removed with a pipette, and the outer surface of each flask was exposed to liquid nitrogen for 1 min to quench the cells. Subsequently, 500 µL of pre‐chilled methanol‐water solution (4:1, v/v) was added, and the cells were scraped off using a cell scraper. Finally, all samples were delivered to Shanghai OE Biotech Co., Ltd for analysis.

### 2.11 Animal Experiments

The animal experiment protocol was approved by the Animal Protection Committee of Wenzhou Institute, University of Chinese Academy of Sciences (Ethics approval number: WIUCAS23092103). All animals were sourced from the Zhejiang Laboratory Animal Center. The biocompatibility of the scaffolds was evaluated in vivo using a subcutaneous implantation model. Male ICR diabetic mice, aged 8–12 weeks, were anesthetized with a mixture of 2% isoflurane and 1% oxygen. The dorsal hair was shaved, and the exposed skin was disinfected with iodine. Under sterile conditions, two 8 mm full‐thickness skin wounds were created on each side of the dorsal skin. The mice were randomly divided into five groups: control (no treatment), base aerogel (Aerogel), amino acid‐loaded aerogel (Aerogel+AA), amino acid and bioactive glass‐loaded aerogel (Aerogel+AA+BC), and a group treated with amino acid, bioactive glass, and thermal therapy‐loaded aerogel (Aerogel+AA+BC+PT), with 20 mice per group. The corresponding 8 mm aerogel samples were applied to each wound, and a silicone fixing ring was sutured using 6‐0 nylon to prevent skin contraction. To protect the wounds from infection, they were covered with sterile dressings (Tegaderm, 3 M, St. Paul, MN, USA). Wound areas were observed daily, and skin samples were collected for histological analysis on days 3, 7, 14, and 21. The wound closure rate (%) was calculated using the following formula: Wound closure rate = (S1 – Sx) / S1 × 100%. Where S1 represents the initial wound area and Sx ​ is the wound area measured on days 3, 7, 14, and 21.

### 2.12 Histological Observations

Skin samples were harvested from the wound sites on days 3, 7, 14, and 21 post‐surgery for histological analysis. The samples were fixed in 4% paraformaldehyde, then dehydrated through a series of graded ethanol solutions, cleared with xylene, and embedded in paraffin. Sections were cut to a thickness of 5 µm and stained using hematoxylin and eosin (HE) for general tissue morphology, and Masson's trichrome staining to assess collagen deposition.

### 2.13 Immunohistochemical Staining

Paraffin‐embedded sections from each experimental group were deparaffinized, rehydrated, and subjected to antigen retrieval. The sections were then blocked with a 5% goat serum BSA solution at room temperature for 1 h, followed by overnight incubation at 4°C with primary antibodies targeting CD45 (1:200), Ly6G (1:200), CCR7 (1:200), CD206 (1:200), IL‐4 (1:200), IL‐10 (1:200), IL‐6 (1:200), and TNF‐α (1:200). After three washes with PBS, the sections were incubated with secondary antibodies at room temperature for 1 h. Visualization was achieved using diaminobenzidine (DAB, 1:20). The sections were subsequently scanned and analyzed using a pathology biopsy scanner.

### 2.14 Statistical Analysis

Statistical data are expressed as mean ± standard deviation (SD). All analyses were conducted using GraphPad Prism software. Group differences were evaluated using one‐way ANOVA, with statistical significance defined as p < 0.05.

## 3 Results and Discussion

### 3.1 Synthesis and Characterization of SrCuSi_4_O_10_ Particles

**FIGURE 1:**
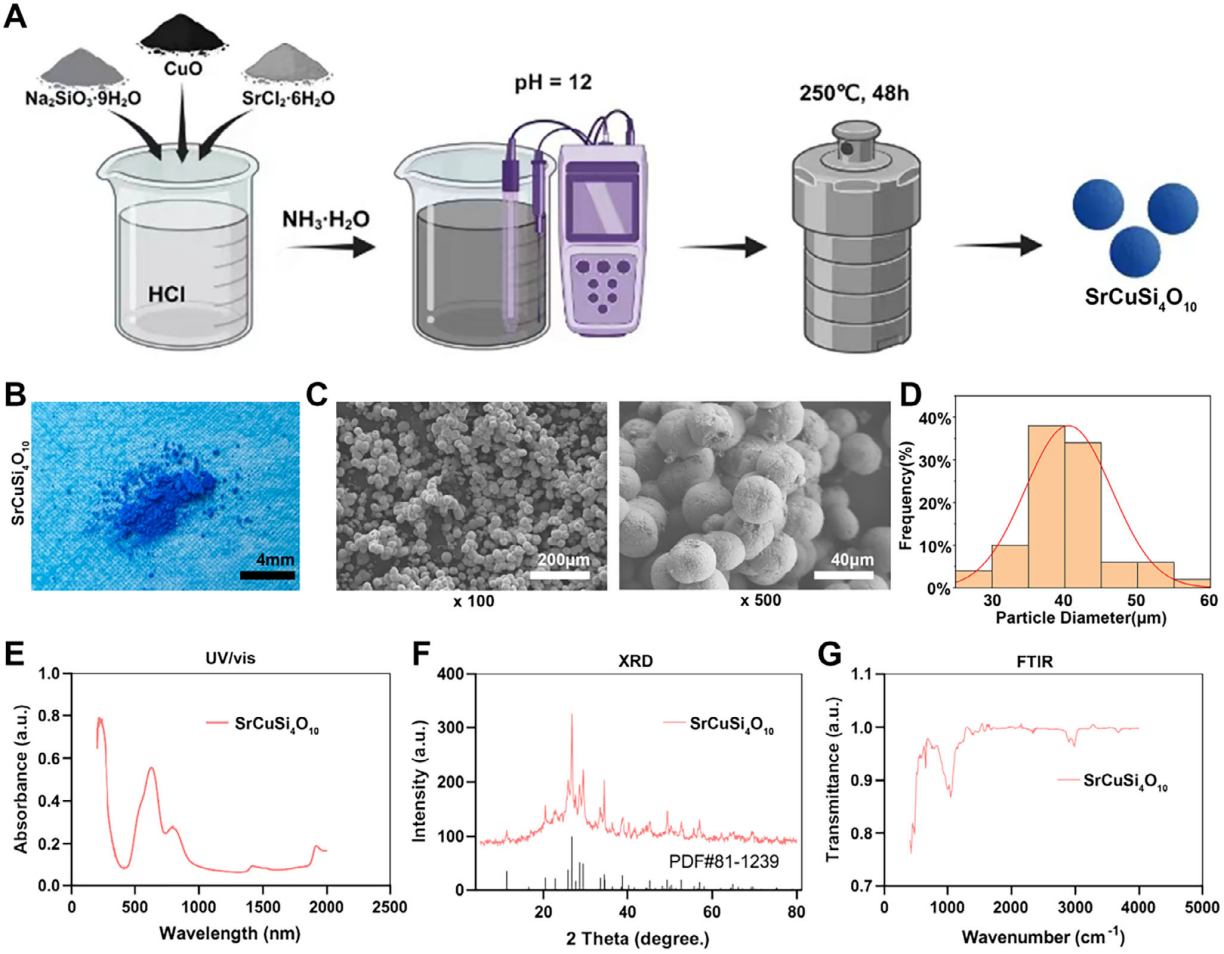
The synthesis and characterization of SrCuSi_4_O_10_ particles. (A) A schematic illustrating the preparation process of SrCuSi_4_O_10_ particles. (B) A photograph of the synthesized SrCuSi_4_O_10_ particles. (C) SEM images showing the morphology of SrCuSi_4_O_10_ particles. (D) Size distribution of the prepared SrCuSi_4_O_10_ particles. (E) UV‐visible absorbance of the developed SrCuSi_4_O_10_ particles. (F) XRD analysis of SrCuSi_4_O_10_ particles. (G) FTIR characterization of SrCuSi_4_O_10_ particles.

Many materials exhibit photothermal effects. In the presented study, we selected bioceramic particles to achieve the “hot spring” effect due to their stable photothermal properties and lower cytotoxicity compared to metal nanoparticles. Moreover, bioceramic particles release inorganic ions that promote tissue repair [21, 22]. As shown in Figure 1A,B, the light blue SrCuSi_4_O_10_ particles were successfully synthesized via a hydrothermal reaction. Figure 1C,D indicated that the SrCuSi_4_O_10_ particles were spherical, with well‐defined morphology, no significant agglomeration, and uniform particle size, with individual particles measuring approximately (40.51 ± 5.89) µm. Figure 1E discovered a strong absorption peak between 500 and 1000 nm was observed, indicating that SrCuSi_4_O_10_ particles could be excited by near‐infrared light (photothermal performance). Figure 1F showed that the SrCuSiO4 particles possessed a complete crystal structure, consistent with the standard card PDF#81‐1239, with strong diffraction peaks at 26.7° and 28.6° and no significant impurity peaks, indicating high particle purity. As shown in Figure 1G, the distinct peaks at Si─O─Si (472 cm^−1^) [23], Cu─O (519 cm^−1^) [24], Sr─O (855 cm^−1^) [25], and Si─O (1048 cm^−1^) confirmed the successful synthesis of SrCuSiO_4_ particles [26].

### 3.2 Fabrication and Characterization of SrCuSi_4_O_10_ Particles and Amino Acids Co‐Loaded Short Nanofiber Aerogel

**FIGURE 2:**
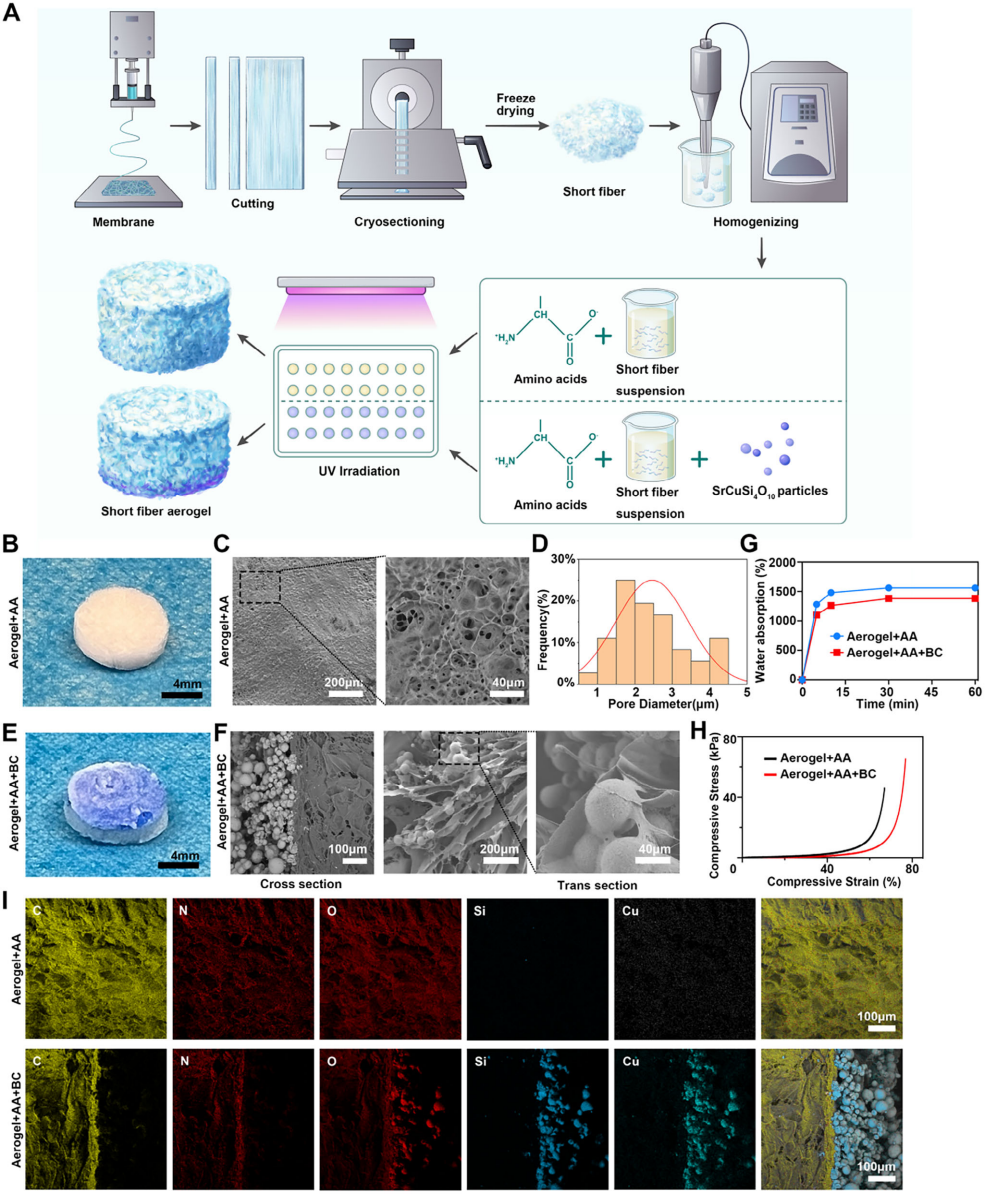
The preparation and characterization of SrCuSi_4_O_10_ particles and amino acids co‐loaded short nanofiber aerogel. (A) The schematic illustrating the preparation processes of SrCuSi_4_O_10_ particles and amino acids co‐loaded short nanofiber aerogel. (B, C) The morphology and internal structure of multiple amino acids loaded short nanofiber aerogel (Aerogel+AA). (D) The internal pore size distribution of amino acids loaded nanofiber aerogel (Aerogel+AA). (E, F) The morphology and internal structure of multiple amino acids and SrCuSi_4_O_10_ particles co‐loaded nanofiber aerogel. (G) PBS absorption behavior of the nanofiber aerogels at room temperature. Water absorption (%) of Aerogel+AA and Aerogel+AA+BC as a function of immersion time (0‐60 min); data are shown as mean values (n = 3).(H) Compressive stress–strain curves of (Aerogel+AA) and (Aerogel+AA+BC) measured on a universal testing machine. (I) The EDX element analysis of the Aerogel+AA and Aerogel+AA+BC.

The PCL/GelMA short nanofiber aerogels were prepared following our previous study [27, 28]. To prepare the SrCuSi_4_O_10_ particles and amino acids co‐loaded short nanofiber aerogel (Aerogel+AA+BC). First, an amino acids mixture solution was prepared by dissolving cysteine, glutamine, lysine, proline, tyrosine, threonine, glutamic acid, and aspartic acid in 0.2% GelMA solution, which play a significant role in cell metabolism (Table 1), which play an important role on cell metabolism [29]. Second, short nanofibers were poured into amino acids mixture solution at the ratio of 0.1 g per 10 mL solution. Finally, the SrCuSi_4_O_10_ particles were added to the mixture and sonicated, then transferred into a home‐designed mold (8 mm in diameter), crosslinked under UV light for 45 s, and freeze‐dried. The small amount of GelMA serves as a crosslinker, creating a network with short fibers, while also acting as a carrier for various amino acids and SrCuSi_4_O_10_ bioceramic particles. Similarly, the multiple amino acids loaded nanofiber aerogel (Aerogel+AA) and nanofiber aerogel without additives were prepared using the same method (Figure 2A).

**TABLE 1:** The role of different amino acids on cell metabolism.

Amino acids	Roles in cell differentiation
Cysteine	Cellular biosynthesis; enzyme catalysis; redox metabolism
Glutamine	Stimulates protein synthesis; reduces ubiquitin‐dependent proteolysis in the enterocyte
Lysine	Key posttranslational modification in cellular regulation; modification of histones and nuclear transcription regulators
Proline	Cell signaling; stress protection; energy production
Tyrosine	Produce hormones such as thyroxine and triiodothyronine; produce neurotransmitters such as dopamine, adrenaline.
Threonine	ATP production; pH regulation.
Glutamic acid	Nitrogen assimilation; amino acid biosynthesis; cofactor production; the production of secondary metabolites such as antibiotics
Aspartic acid	Maintaining intracellular redox balance; nucleotide synthesis; TCA cycle anaplerosis

The aerogel loaded with only amino acids exhibits a white (Figure 2B), porous structure with internal pore sizes ranging from 0.5 to 4.5 µm (Figure 2B,C). The aerogel mixed with SrCuSi_4_O_10_ bioceramic and amino acids appears light blue (Figure 2E), with the bottom of the aerogel showing a distinct blue color. SEM analysis further revealed that the SrCuSi_4_O_10_ bioceramic‐loaded aerogel is divided into two parts. The upper layer contains a small amount of SrCuSi_4_O_10_ bioceramic and a large amount of short fibers, while the lower layer primarily consists of SrCuSi_4_O_10_ bioceramic with a small amount of short fibers (Figure 2F). Both aerogels showed rapid PBS uptake at room temperature, reaching a plateau by 30 min (Aerogel+AA: 1560%; Aerogel+AA+BC: 1380%). Incorporation of SrCuSi_4_O_10_ bioceramic particles slightly reduced the absorption capacity but did not alter the fast‐uptake kinetics (Figure 2G). Both aerogels showed foam‐like, J‐shaped stress–strain curves—low stress at small strains with a steep rise beyond ∼60%. Aerogel+AA+BC was slightly less stiff than Aerogel+AA yet remained stable to 80% strain. Peak stresses were in the tens of kPa (Figure 2H). Elemental mapping analysis (Figure 2I) provides further insights into the composition of both aerogels. In Aerogel+AA, the elemental mapping shows a uniform distribution of C, N, and O. In contrast, the composite aerogel (Aerogel+AA+BC) displays the presence of silicon (Si) and copper (Cu), verifying the successful integration of SrCuSi_4_O_10_ particles into the aerogel structure.

### 3.3 Evaluation of the Photothermal Effect of SrCuSi_4_O_10_ Particles Loaded Short Nanofiber Aerogels

**FIGURE 3:**
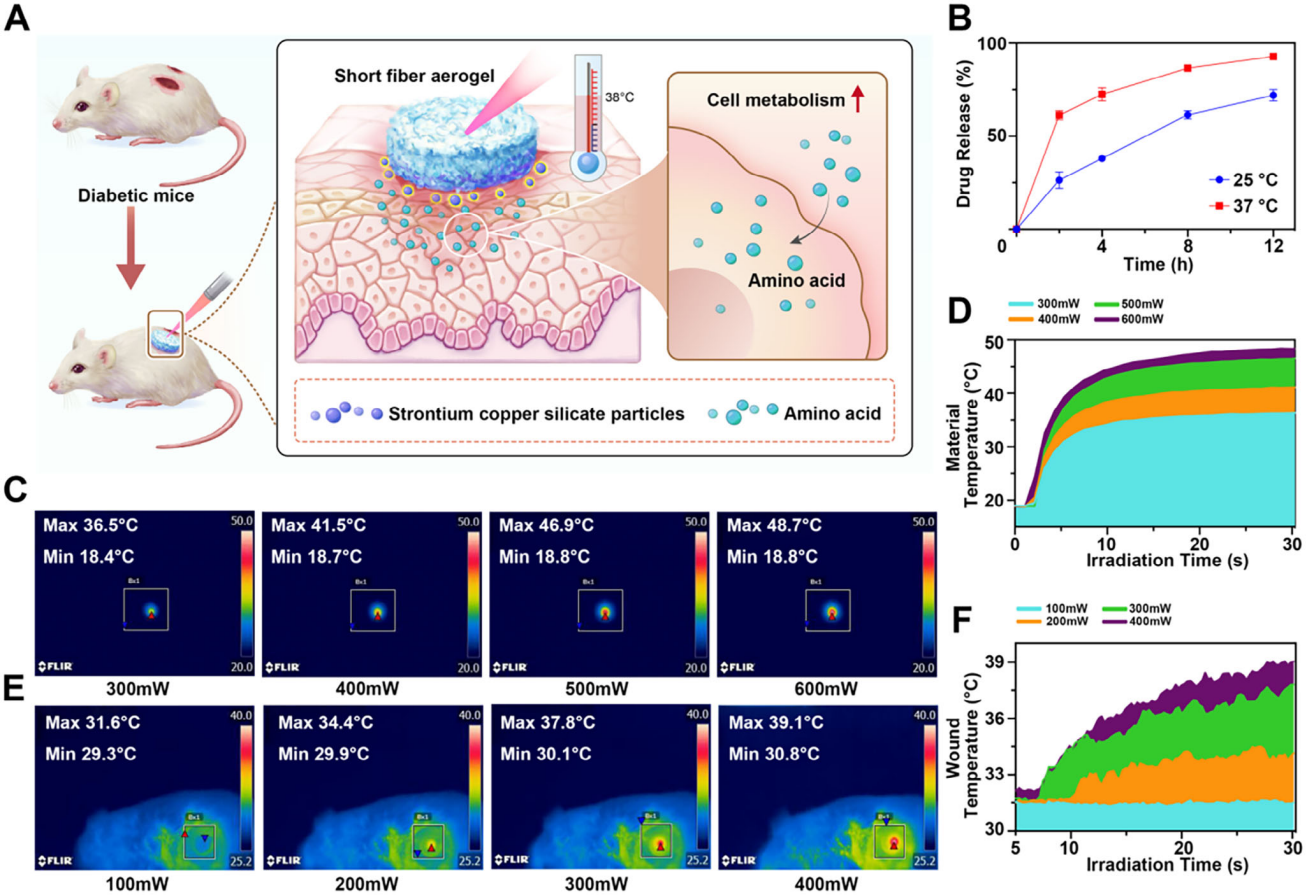
The SrCuSi_4_O_10_ particles and amino acids co‐loaded nanofiber aerogel act as hot spring therapy, promoting amino acid absorption and cell metabolism. (A)The schematic illustrating the application of SrCuSi4O10 particles and amino acids co‐loaded nanofiber aerogel on diabetic wound healing, the SrCuSi4O10 particles exert photothermal effects and increase local temperature, thereby promoting the body's absorption of amino acids and promoting cell metabolism. (B) Cumulative amino‐acid release from the aerogel at 25°C and 37°C in PBS (pH 7.4); release is faster at 37°C and nears completion by 12 h (mean ± SD, n = 3). (C, D) The photothermal effect of SrCuSi4O10 particles under different irradiation conditions. The laser power ranged from 300 to 600 mW (E, F) The photothermal behaviors of SrCuSi4O10 particles after subcutaneous implantation under different irradiation conditions. The laser power ranged from 100 to 400 mW.

First, We examined the release profile of amino acid–loaded aerogels under different temperature conditions. As shown in Figure 3B, amino acid release was both time‐ and temperature‐dependent. At 37°C, the aerogel displayed a biphasic release pattern, characterized by an initial rapid burst phase within the first 0–4 h, followed by a slower diffusion‐controlled stage, with cumulative release reaching nearly complete levels by approximately 12 h. At 25°C, a similar trend was observed, but the release kinetics were markedly slower, resulting in a lower cumulative fraction at the same time point. The accelerated release at 37°C can be attributed to enhanced matrix hydration and increased polymer chain mobility, which collectively facilitate amino acid diffusion. These results confirm that the photothermal effect enables more efficient and timely delivery of amino acids under physiologically relevant conditions.

We investigated the in vitro photothermal effects of bioactive glass. As shown in Figure 3C,D, we found that a minimum of 300 mW of radiant power is required to induce the photothermal effect of bioceramic in vitro. After 30 s of irradiation at 300 mW and 400 mW, the material's temperature reached 36°C and 40°C, respectively. Additionally, as the radiation power increases, the temperature generated by the bioceramic also rises. In vivo, when the bioceramic is irradiated at 400 mW for 30 s, the central temperature can reach 40°C, with the surrounding temperature remaining stable between 36°C and 40°C (Figure 3E,F), This temperature range is slightly above normal body temperature yet well below the threshold for thermal injury, providing a safe hyperthermic stimulus that enhances cellular activity and immune responses [30, 31, 32].

### 3.4 Physiological Temperature Enhances Amino‐Acid–Mediated Fibroblast Migration Without Cytotoxicity

**FIGURE 4:**
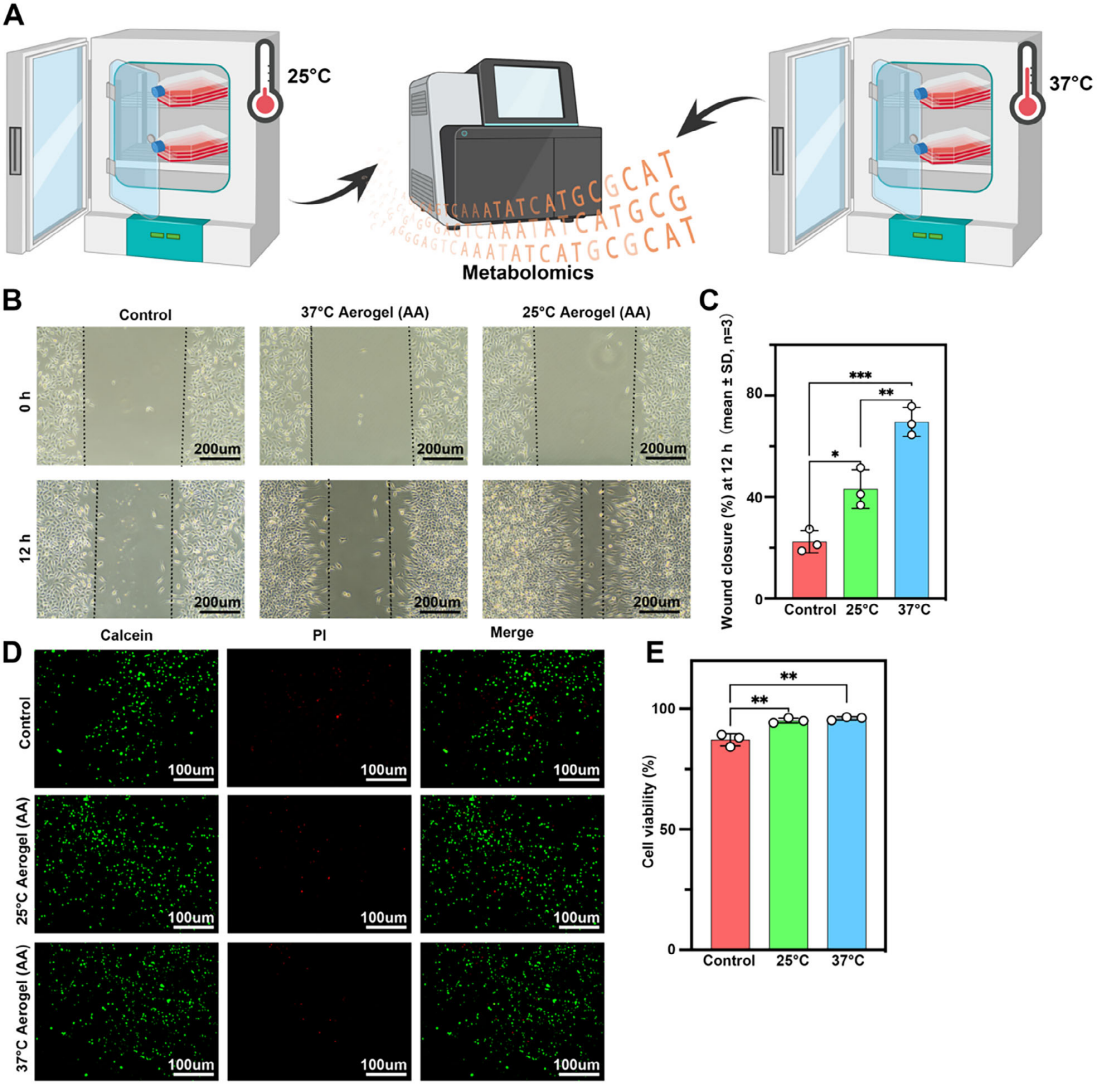
In vitro assessment of temperature‐regulated amino‐acid effects on fibroblasts. (A) The schematic illustrates primary fibroblasts were treated with multiple amino acids and cultured at 25°C and 37°C for metabolomic analysis, respectively. (B) Wound‐healing (scratch) assay micrographs at 0 h and 12 h for control medium and for media conditioned with amino‐acid–loaded aerogels prepared at 25°C or 37°C; dashed lines denote the initial wound edges (scale bars, 200 µm). (C) Quantification of fibroblast migration in the scratch (wound‐healing) assay. Wound closure (%) at 12 h for the Control, 25°C Aerogel (AA), and 37°C Aerogel (AA) groups. (D) Live/dead staining after 24 h in the same media showing Calcein‐AM (live, green) and propidium iodide, PI (dead, red) (scale bars, 100 µm). Representative of ≥3 independent experiments. (E) Quantitative analysis of cell viability based on Live/Dead staining. Cell viability (%) for the Control, 25°C Aerogel (AA), and 37°C Aerogel (AA) groups after 24 h incubation.

We aim to use bioceramic and amino acids co‐loaded nanofiber aerogels for chronic wound repair. The photothermal effect of bioceramic can increase the local temperature of the wound, promoting the body's absorption of amino acids and accelerating cell metabolism (Figure 4A). To verify this hypothesis, we first cultured fibroblasts in vitro at 25°C and 37°C and added various amino acids to the culture medium. Finally, we used metabolomics to assess whether the higher temperature of 37°C could promote amino acid absorption and enhance cell metabolism more effectively than 25°C (Figure 4A). In the scratch assay (Figure 4B,C), the 37°C AA group exhibited visibly faster gap closure within 12 h than either the 25°C AA group or the control medium, indicating enhanced migratory activity. Quantitative analysis confirmed a significantly higher wound‐closure rate at 12 h in the 37°C AA group compared with the 25°C AA and control groups (mean ± SD, *n* = 3; one‐way ANOVA with Tukey's post hoc test).Live/dead staining after 24 h showed predominantly Calcein‐positive cells and sparse PI‐positive nuclei across all groups (Figure 4D,E), confirming low cytotoxicity; cell density was highest in the 37°C AA condition. Consistently, quantification of cell viability revealed no obvious cytotoxicity among groups, with viability remaining above ∼90% (mean ± SD, *n* = 3).Collectively, these data show that maintaining physiological temperature augments the pro‐migratory effect of the amino‐acid cocktail without compromising viability, motivating subsequent metabolomic analysis to define the underlying pathways. A limitation of this study is that the cellular‐level metabolic effects of Aerogel+AA+BC were not directly quantified. Although extract‐based proliferation and migration assays provide indirect evidence of its bioactivity, they cannot fully resolve whether Aerogel+AA+BC induces specific intracellular metabolic reprogramming. Future work will incorporate targeted and/or untargeted metabolomic analyses and pathway‐focused assays to directly define the metabolic mechanisms involved.

### 3.5 The “Hot Spring” Effect Is Beneficial in Promoting the Absorption and Metabolism of Amino Acids by Cells

**FIGURE 5:**
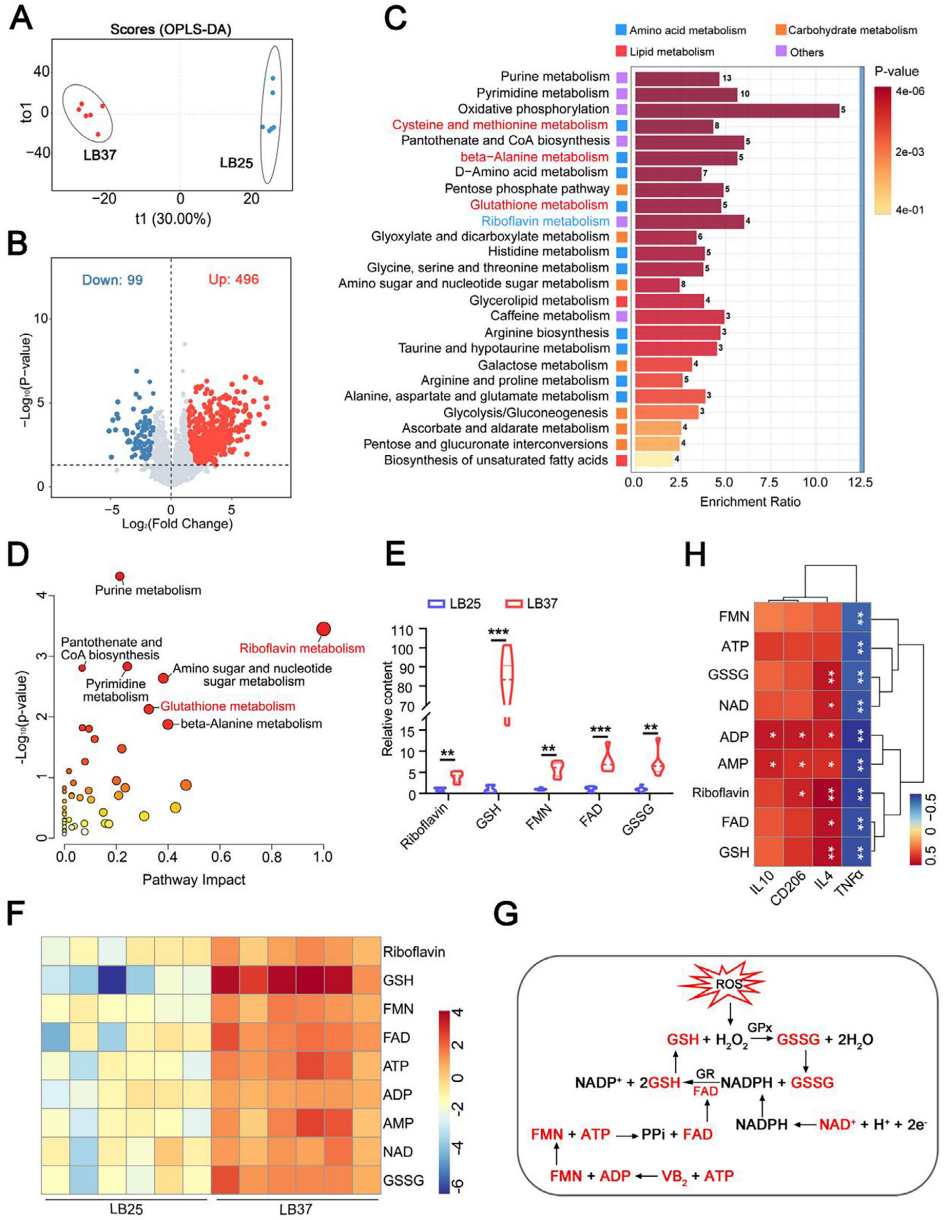
Metabolomic analysis shows significant activation of the glutathione redox reaction in the multiple amino acids treated group under 37°C. (A) OPLS‐DA analysis presents a score plot of metabolic separation between the 25°C and 37°C groups. (B) Volcano plot shows the differential metabolites between the 25°C and 37°C groups. (C) KEGG enrichment analysis displays the top 25 significantly different metabolic pathways. (D) Metabo Analyst pathway analysis of differential metabolites between the two groups. The vertical axis represents the p‐value from pathway enrichment analysis (expressed as a negative logarithm), and the horizontal axis indicates the impact factor from pathway topology analysis. Larger circle diameters indicate a higher impact factor, and deeper red color signifies a greater ‐log [10] (p‐value), indicating a higher degree of enrichment. (E, F) Bar plots and heatmaps show the relative changes of differential metabolites in riboflavin metabolism and glutathione metabolism pathways. (G) Schematic diagram of the glutathione redox reaction. Red markers indicate metabolites with significant differences in this experiment. GPx and GR represent glutathione peroxidase and glutathione reductase, respectively. (H) Heatmap analysis of the correlation between glutathione redox reaction‐related metabolites and inflammatory factors. LB25: 25°C. LB37:37°C. **p<0.01, ***p<0.001.

To explore the metabolic changes in fibroblasts under different temperature conditions and to identify key metabolites, we conducted a metabolomic analysis of the 25°C and 37°C groups using a dual platform based on LC‐MS/GC‐MS. The results showed that the OPLS‐DA model successfully distinguished between the two groups (Figure 5A). A total of 2,755 metabolites were identified. After filtering out metabolites detected in fewer than 3 individuals per group, 2,322 metabolites remained, of which 595 showed statistically significant differences (*p* < 0.05, VIP > 1) (Table S1). Compared to 25°C, 496 metabolites were significantly increased in 37°C, while the remaining metabolites decreased (Figure 5B). KEGG enrichment analysis indicated that the differential metabolites were primarily concentrated in amino acid metabolism pathways, including cysteine and methionine metabolism, β‐alanine metabolism, and glutathione metabolism (Figure 5C). Based on p‐values and pathway impact scores, riboflavin metabolism and glutathione metabolism were selected for further study (Figure 5D). Quantitative analysis of the differential metabolites in these two pathways revealed significant increases in riboflavin, glutathione (GSH), flavin mononucleotide (FMN), flavin adenine dinucleotide (FAD), and glutathione disulfide (GSSG) in the 37°C group (Figure 5E). Additionally, significant changes were observed in adenosine triphosphate (ATP), adenosine diphosphate (ADP), adenosine monophosphate (AMP), and nicotinamide adenine dinucleotide (NAD), all of which are involved in the glutathione redox reaction. Riboflavin metabolism provides the necessary cofactors for this reaction (Figure 5F,G). Further correlation analysis revealed that metabolites such as GSH (reduced glutathione), GSSG (oxidized glutathione), and riboflavin were positively correlated with the anti‐inflammatory factor IL‐4, IL‐10 and negatively correlated with the pro‐inflammatory factor TNFα (Figure 5H). This suggests that activating the glutathione redox reaction in the multiple amino acids treated cells under 37°C could enhance the cells’ ability to resist oxidative stress and anti‐inflammation [33, 34], which can change the inflammatory microenvironment of the chronic wound bed and bring the wound healing into a normal healing process [35].

### 3.6 In Vivo Wound Healing

**FIGURE 6:**
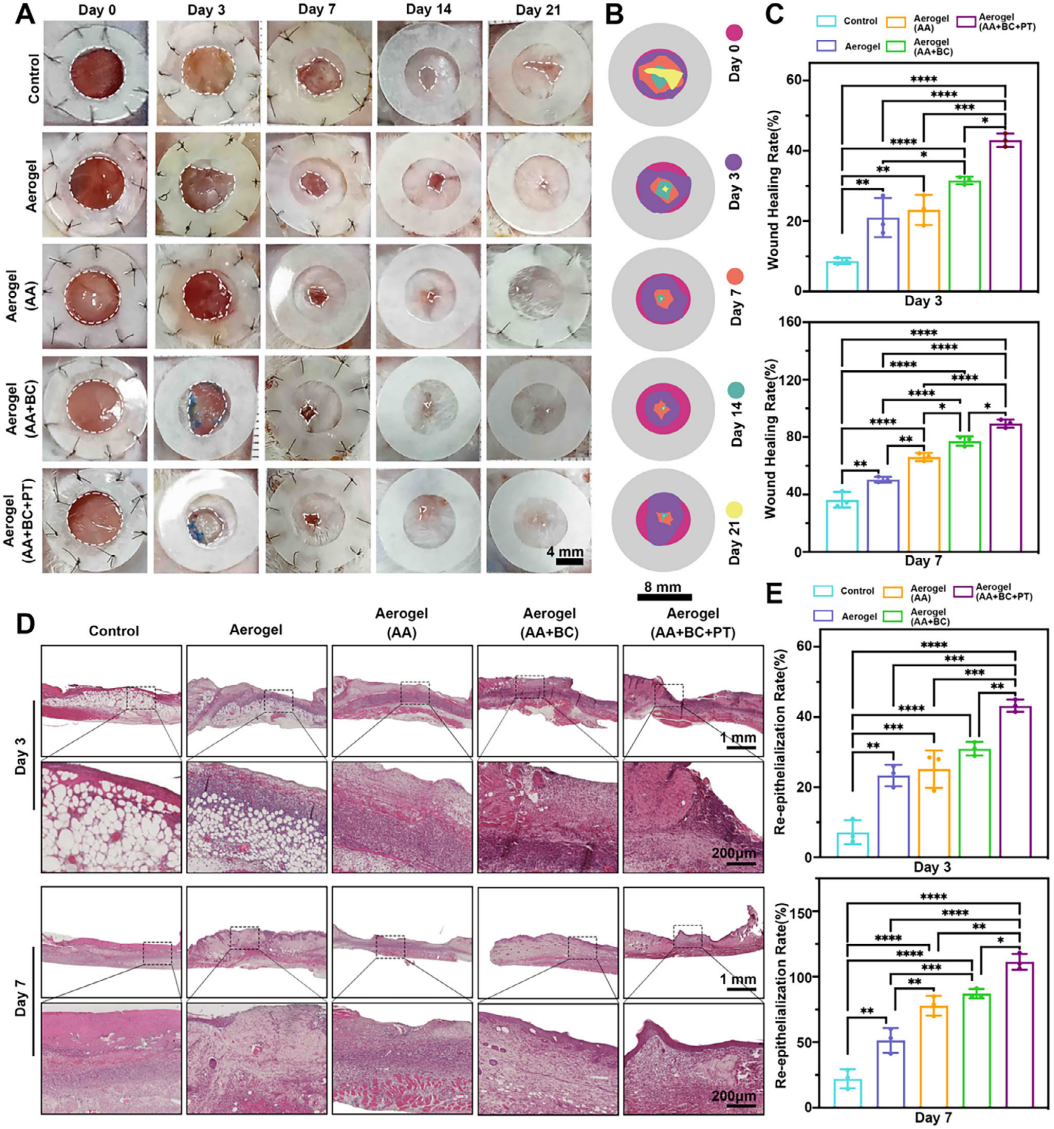
The composited aerogel in combination with photothermal therapy promotes diabetic wound healing. (A) The photographs of the wound treated with aerogel, aerogel (AA), aerogel (AA+BC), and aerogel (AA+BC+PT) groups for 3, 7, 14, and 21 days. The wounds without any treatment as control. (B) The schematic illustrates the changes in skin wound size of each group during wound healing. (C) The comparison of wound healing rates between groups after 3 and 7 days of treatment. (D) The histological observations of the whole wound area treated with aerogel, aerogel (AA), aerogel (AA+BC), and aerogel (AA+BC+PT) groups for 3 and 7 days. (E) The re‐epithelialization rate of the wounds treated with aerogel, aerogel (AA), aerogel (AA+BC), and aerogel (AA+BC+PT) groups for 3 and 7 days. *p<0.05, **p<0.01, ***p<0.001, ****p<0.0001.

In each mouse, an 8‐mm‐diameter wound was created and treated with various materials, including aerogel, amino acid‐loaded aerogel (Aerogel+AA), bioceramic and amino acid co‐loaded aerogel (Aerogel+AA+BC), and bioceramic and amino acid co‐loaded aerogel combined with photothermal therapy (Aerogel+AA+BC+PT). During irradiation, the wound temperature increased and stabilized between 30°C and 40°C. Each session lasted 30 s, followed by a 30‐s pause, for a total of 10 min, administered twice daily for 7 days. Wound healing progression was documented with images taken on days 0, 3, 7, 14, and 21 (Figure 6A). The healing rate was significantly higher in the amino acid‐loaded aerogel group compared to the group without amino acids. Moreover, wounds treated with both amino acid‐loaded aerogel and photothermal therapy healed faster, especially on day 3 (Figure 6A–C).

**FIGURE 7:**
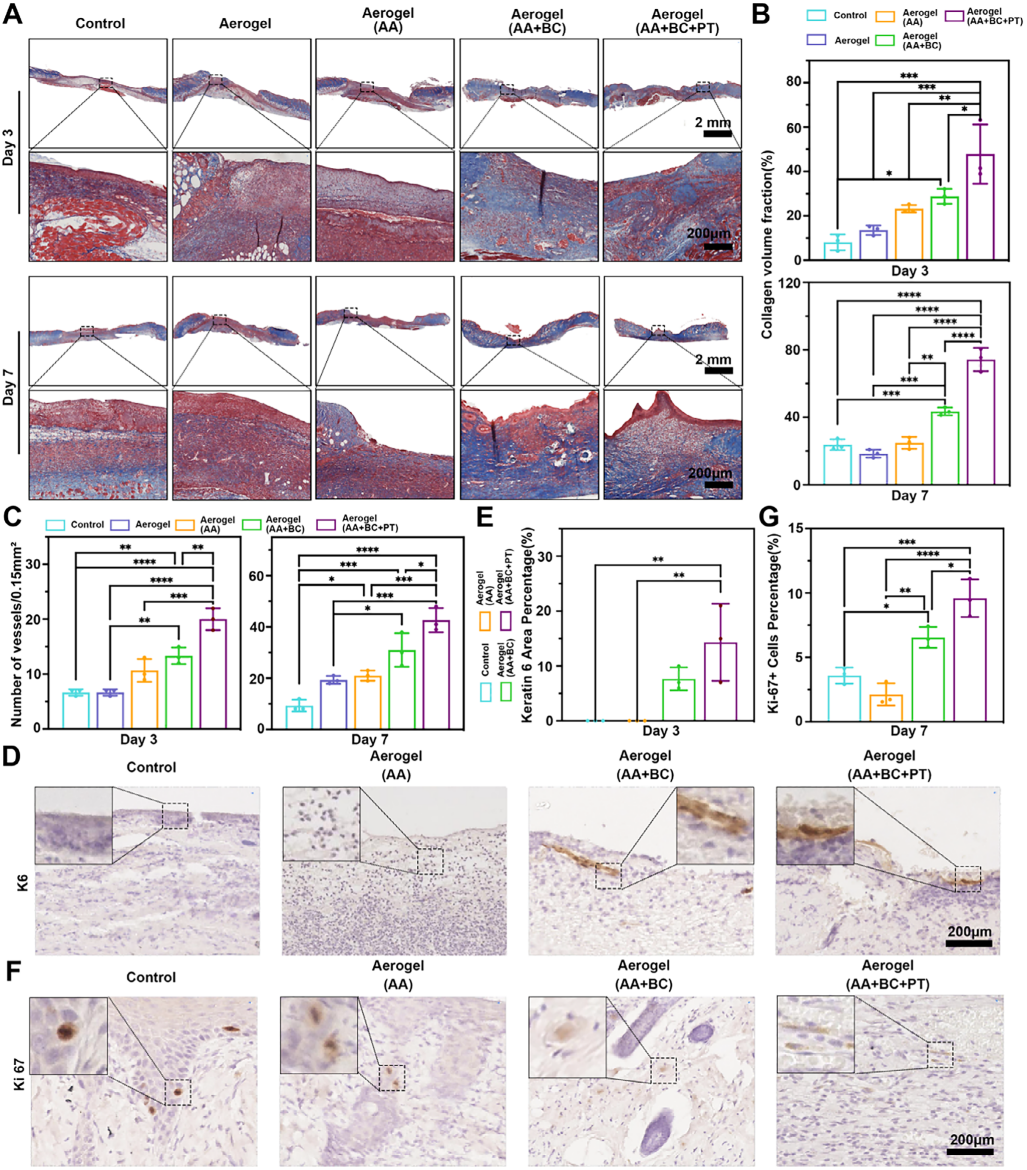
Evaluation of activities in the proliferative phase of wound healing. (A) The Masson trichrome staining of the whole wound area was treated with aerogel, aerogel (AA), aerogel (AA+BC), and aerogel (AA+BC+PT) groups for 3 and 7 days. (B) The quantification of collagen deposition in the wounds treated with aerogel, aerogel (AA), aerogel (AA+BC), and aerogel (AA+BC+PT) groups for 3 and 7 days. (C) The quantification of newly formed blood vessels within the wound of aerogel, aerogel (AA), aerogel (AA+BC), and aerogel (AA+BC+PT) groups on day 3 and day 7. (D, E) The immunohistochemical staining and quantification of keratin 6 (K6) at the wound edge area of aerogel, aerogel (AA), aerogel (AA+BC), and aerogel (AA+BC+PT) groups on day 7. (F, G) The immunohistochemical staining and quantification of KI67 at the wound edge area of aerogel, aerogel (AA), aerogel (AA+BC), and aerogel (AA+BC+PT) groups on day 7. *p<0.05, **p<0.01, ***p<0.001, ****p<0.0001.

H&E staining revealed that on day 3, the Aerogel+AA+BC+PT group showed accelerated re‐epithelialization, with a thicker epithelial layer and a smaller wound gap. By day 7, nearly complete re‐epithelialization was observed in this group, with well‐organized tissue, indicating faster wound regeneration compared to the control and other aerogel‐treated groups (Figure 6D). Quantitative analysis confirmed that the Aerogel+AA+BC+PT group had the highest re‐epithelialization rate by day 3, nearing 100% by day 7, significantly outpacing other treatments (Figure 6E). Masson trichrome staining assessed collagen deposition on days 3 and 7. Wounds treated with Aerogel+AA+BC+PT displayed denser, more organized collagen fibers compared to the control and other treatment groups. This trend persisted through day 7, where the Aerogel+AA+BC+PT group exhibited significantly enhanced collagen deposition (Figure 7A). Quantitative analysis showed that collagen volume was markedly higher in this group on both days 3 and 7 (Figure 7B). Neovascularization was measured by counting newly formed blood vessels in the wound sections (Figure 7C). On days 3 and 7, the Aerogel+AA+BC+PT group had a significantly higher number of newly formed vessels than the control and other groups.

Epidermal hyperplasia and granulation tissue formation are the most important activities in the proliferative phase of wound healing [36]. Epidermal hyperproliferation at the wound edge was evaluated through keratin 6 (K6) expression, indicating that keratinocytes have strong proliferation activity [37]. On day 3, the Aerogel+AA+BC+PT group exhibited significantly elevated K6 expression, indicating enhanced keratinocyte proliferation. By day 7, K6 expression increased further, showing more advanced re‐epithelialization than the control group (Figure 7D,F). Cell proliferation within granulation tissue was assessed using Ki‐67 staining. On day 7, the percentage of Ki‐67 positive cells was measured. The Aerogel+AA+BC+PT group showed an increased Ki‐67 expression compared to the other 4 groups (Figure 7E,G). Suggesting the treatment of Aerogel+AA+BC+PT could enhance the proliferative phase activities of wound healing, thereby promoting skin tissue defect repair and wound closure.

### 3.7 Evaluation of Inflammatory Responses

**FIGURE 8:**
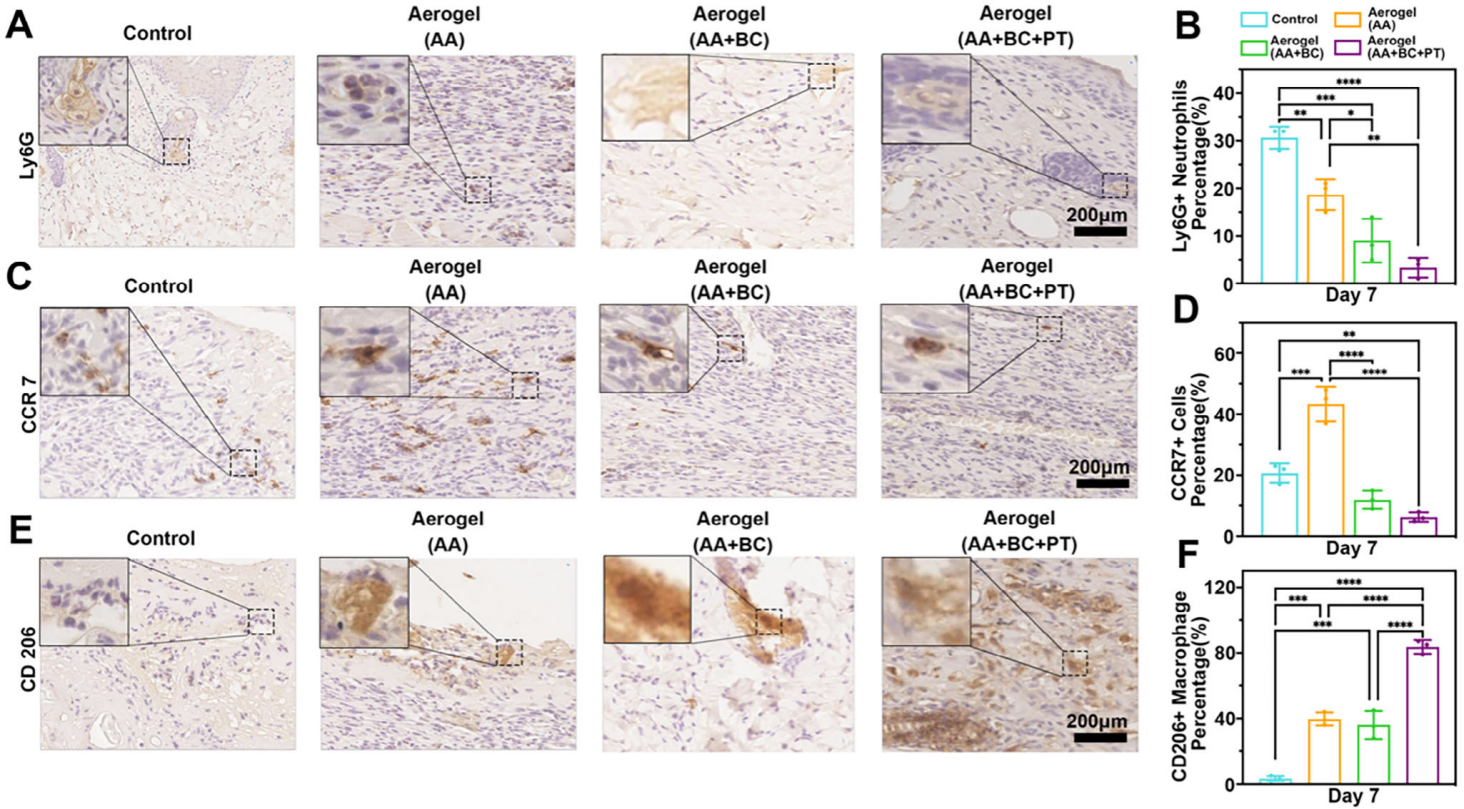
The expression of inflammatory cells in the wound area of control, aerogel (AA), aerogel (AA+BC), and aerogel (AA+BC+PT) groups during wound healing. (A, B) The expression and quantification of Ly6G in the wound area of control, aerogel (AA), aerogel (AA+BC), and aerogel (AA+BC+PT) groups on day 7. (C, D) The expression and quantification of CCR7 in the wound area of control, aerogel (AA), aerogel (AA+BC), and aerogel (AA+BC+PT) groups on day 7. (E, F) The expression and quantification of CD206 in the wound area of control, aerogel (AA), aerogel (AA+BC), and aerogel (AA+BC+PT) groups on day 7. *p<0.05, **p<0.01, ***p<0.001, ****p<0.0001.

**FIGURE 9:**
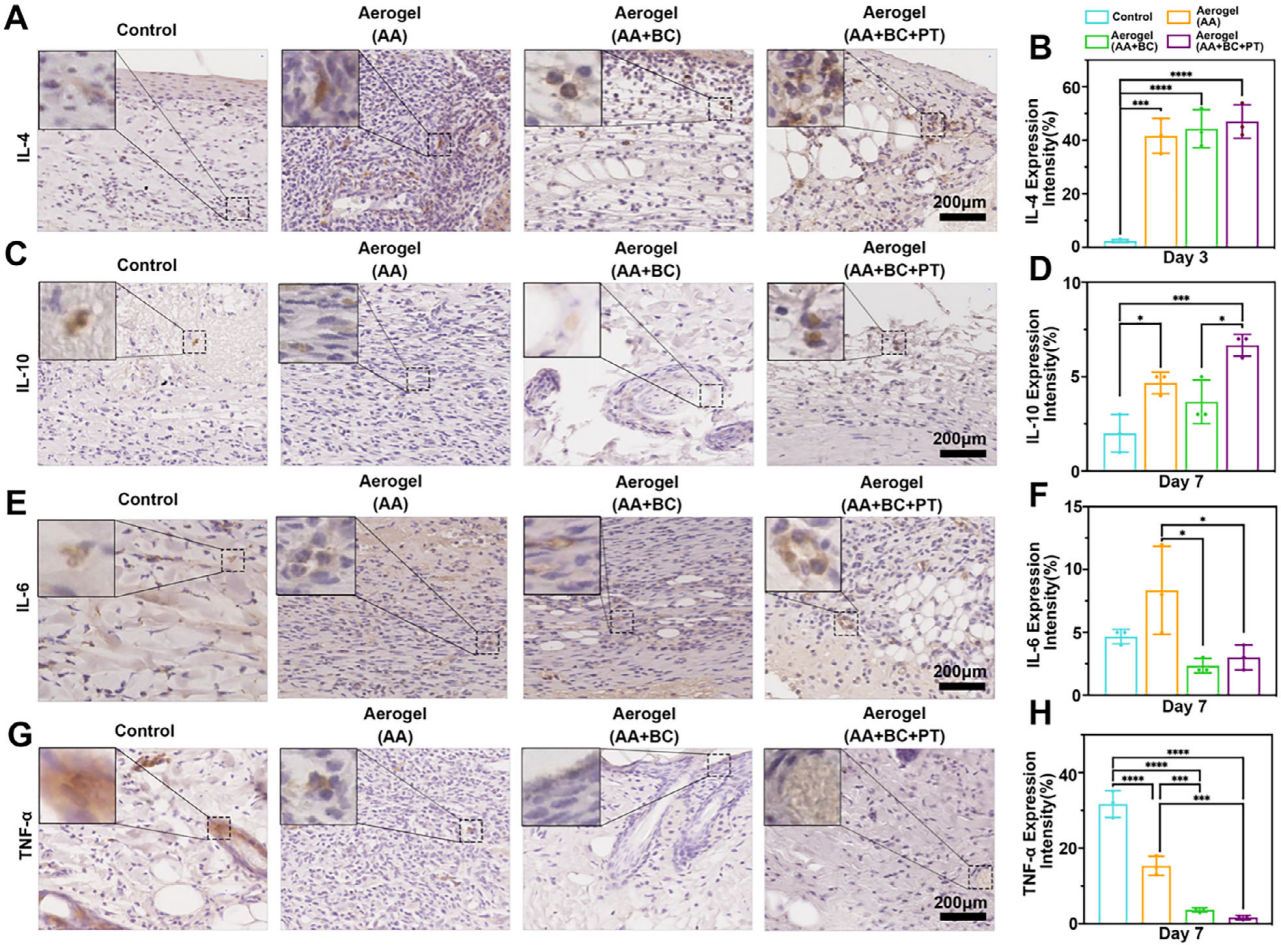
The expression of inflammatory factors in the wound area of control, aerogel (AA), aerogel (AA+BC), and aerogel (AA+BC+PT) groups during wound healing. (A, B) The expression and quantification of IL‐4 in the wound area of control, aerogel (AA), aerogel (AA+BC), and aerogel (AA+BC+PT) groups on day 7. (C, D) The expression and quantification of IL‐10 in the wound area of control, aerogel (AA), aerogel (AA+BC), and aerogel (AA+BC+PT) groups on day 7. (E, F) The expression and quantification of IL‐6 in the wound area of control, aerogel (AA), aerogel (AA+BC), and aerogel (AA+BC+PT) groups on day 7. (G, H) The expression and quantification of TNF‐α in the wound area of control, aerogel (AA), aerogel (AA+BC), and aerogel (AA+BC+PT) groups on day 7. *p<0.05, **p<0.01, ***p<0.001, ****p<0.0001.

Previous metabolomic experiments have confirmed that increasing the temperature enhances the cellular metabolism of various amino acids, particularly by boosting the glutathione redox reaction, which plays a key role in anti‐inflammatory processes [38]. Therefore, we further verified the infiltration of inflammatory cells and the expression of inflammatory factors in vivo. After 7 days of treatment, wounds treated with Aerogel+AA+BC+PT showed significantly reduced neutrophil infiltration, indicated by lower Ly6G expression, compared to the control and other aerogel‐treated groups (Figure 8A,B). Macrophage polarization was evaluated using CCR7 to mark pro‐inflammatory M1 macrophages and CD206 to identify anti‐inflammatory M2 macrophages. On day 7, the Aerogel+AA+BC+PT group had significantly fewer CCR7‐positive macrophages than the other groups (Figure 8C,D), while CD206 expression was markedly higher, suggesting a shift from a pro‐inflammatory to a regenerative wound environment (Figure 8E,F). In addition, the expression of inflammatory cytokines was assessed. On day 7, anti‐inflammatory cytokines IL‐4 and IL‐10 were significantly elevated in the Aerogel+AA+BC+PT‐treated wounds compared to the control group (Figure 9A–D). In contrast, pro‐inflammatory cytokines IL‐6 and TNF‐α were downregulated in the Aerogel+AA+BC+PT group relative to the control and other aerogel‐treated groups (Figure 9E–H). The in vivo animal experiment results were consistent with the findings from in vitro metabolomic analysis. In vivo mechanistic evidence in the current work is mainly derived from histology and immunohistochemistry, which provides spatial information but limited quantitative depth. We will include complementary molecular assays (e.g., WB, qPCR, and/or ELISA) in follow‐up studies to strengthen the robustness of the proposed mechanism.

## 4 Conclusion

**FIGURE 10:**
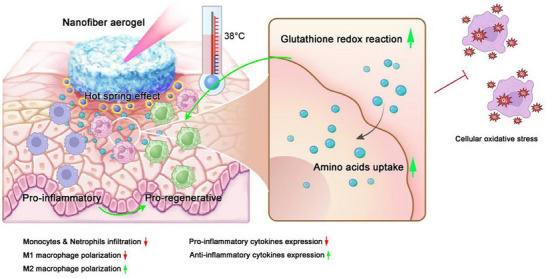
The potential mechanism of the SrCuSi_4_O_10_ particles and multiple amino acids co‐loaded nanofiber aerogel promoting diabetic wound healing. The SrCuSi_4_O_10_ bioceramic particles mimic the effects of hot spring therapy by increasing the local wound temperature, which enhances the absorption of amino acids and stimulates cell metabolism. The amino acid uptake supports cellular glutathione redox reactions, helping to mitigate oxidative stress. Additionally, this process shifts the local inflammatory response from a pro‐inflammatory to a pro‐regenerative state, creating an optimal wound bed for the repair of diabetic chronic wounds.

This study proposes a new chronic wound treatment plan from the perspective of enhancing the cell metabolism of chronic wound. To prove our concept, we developed an extracellular matrix‐mimicking nanofiber aerogel co‐loaded with eight essential amino acids and SrCuSi_4_O_10_ bioceramic particles. The “hot spring” effect produced by SrCuSi_4_O_10_ bioceramic particles can promote the uptake of exogenous amino acids, thereby enhancing cell metabolism. First, the in vitro studies with primary fibroblasts cultured at 25°C and 37°C revealed significant activation of the glutathione redox pathway in the amino acid‐treated group at 37°C, which can help to mitigate oxidative stress and enhance anti‐inflammation. In vivo, the combination of amino acid‐loaded aerogels and SrCuSi_4_O_10_ particles, along with photothermal therapy, significantly promoted wound closure, cell proliferation, collagen deposition, and angiogenesis. Immunohistochemical analysis showed a reduction in neutrophil infiltration, M1 macrophage polarization, and pro‐inflammatory cytokines, while increasing M2 macrophage polarization and anti‐inflammatory cytokine production, thus shifting the local inflammatory response from pro‐inflammatory to regenerative (Figure 10). The in vivo animal experiment results align with the findings from in vitro metabolomic analysis. This approach demonstrates potential as an effective therapeutic option for enhancing the healing of chronic wounds. In future work, we will systematically summarize heat‐based wound therapies and compare them with our photothermal aerogel approach to clarify potential advantages in temperature controllability and localized, on‐demand treatment.

## Conflicts of Interest

The authors declare no conflicts of interest.

## Data Availability

The data that support the findings of this study are available from the corresponding author upon reasonable request.

## References

[1] K. Hamidzadeh , S. M. Christensen , E. Dalby , P. Chandrasekaran , and D. M. Mosser , “Macrophages and the Recovery from Acute and Chronic Inflammation,” Annual Review of Physiology 79, no. 1 (2017): 567–592, 10.1146/annurev-physiol-022516-034348. PMC5912892 27959619

[2] P. Martin and R. Nunan , “Cellular and Molecular Mechanisms of Repair in Acute and Chronic Wound Healing,” British Journal of Dermatology 173, no. 2 (2015): 370–378, 10.1111/bjd.13954. 26175283 PMC4671308

[3] O. A. Peña and P. Martin , “Cellular and Molecular Mechanisms of Skin Wound Healing,” Nature Reviews Molecular Cell Biology 25 (2024): 599–616. 38528155 10.1038/s41580-024-00715-1

[4] X. Zhao , D. Pei , Y. Yang , et al., “Green Tea Derivative Driven Smart Hydrogels with Desired Functions for Chronic Diabetic Wound Treatment,” Advanced Functional Materials 31, no. 18 (2021): 2009442, 10.1002/adfm.202009442.

[5] Y. Xiong , X. Chu , T. Yu , et al., “Reactive Oxygen Species‐Scavenging Nanosystems in the Treatment of Diabetic Wounds,” Advanced Healthcare Materials 12 (2023): 2300779, 10.1002/adhm.202300779. 37051860

[6] S. A. Eming , T. A. Wynn , and P. Martin , “Inflammation and Metabolism in Tissue Repair and Regeneration,” Science 356, no. 6342 (2017): 1026–1030, 10.1126/science.aam7928. 28596335

[7] K. Palikaras , E. Lionaki , and N. Tavernarakis , “Balancing Mitochondrial Biogenesis and Mitophagy to Maintain Energy Metabolism Homeostasis,” Cell Death & Differentiation 22, no. 9 (2015): 1399–1401, 10.1038/cdd.2015.86. 26256515 PMC4532782

[8] S. A. Eming , P. J. Murray , and E. J. Pearce , “Metabolic Orchestration of the Wound Healing Response,” Cell Metabolism 33, no. 9 (2021): 1726–1743, 10.1016/j.cmet.2021.07.017. 34384520

[9] T. W. Mak , M. Grusdat , G. S. Duncan , et al., “Glutathione Primes T Cell Metabolism for Inflammation,” Immunity 46 (2017): 675–689. 28423341 10.1016/j.immuni.2017.03.019

[10] G. Morris , M. Gevezova , V. Sarafian , and M. Maes , “Redox Regulation of the Immune Response,” Cellular & Molecular Immunology 19, no. 10 (2022): 1079–1101, 10.1038/s41423-022-00902-0. 36056148 PMC9508259

[11] Z. Wang , F. Zhao , C. Xu , et al., “Metabolic Reprogramming in Skin Wound Healing,” Burns & Trauma 12 (2024): tkad047, 10.1093/burnst/tkad047. 38179472 PMC10762507

[12] Y. Zhang , C. B. Higgins , S. Tica , et al., “Hierarchical Tricarboxylic Acid Cycle Regulation by Hepatocyte Arginase 2 Links the Urea Cycle to Oxidative Metabolism,” Cell metabolism 36, no. 9 (2024): 2069–2085.e8, 10.1016/j.cmet.2024.07.007.39116884

[13] J. Gambardella , W. Khondkar , M. B. Morelli , X. Wang , G. Santulli , and V. Trimarco , “Arginine and Endothelial Function,” Biomedicines 8, no. 8 (2020): 277. 32781796 10.3390/biomedicines8080277 PMC7460461

[14] G. J. McBean , “Cysteine, Glutathione, and Thiol Redox Balance in Astrocytes,” Antioxidants 6 (2017): 62. 28771170 10.3390/antiox6030062 PMC5618090

[15] G. Gethin , J. D. Ivory , D. Sezgin , H. Muller , G. O'Connor , and A. Vellinga , “What Is the “Normal” Wound Bed Temperature? A Scoping Review and New Hypothesis,” Wound Repair and Regeneration 29, no. 5 (2021): 843–847, 10.1111/wrr.12930. 33987906

[16] D. G. Armstrong , K. Holtz‐Neiderer , C. Wendel , M. J. Mohler , H. R. Kimbriel , and L. A. Lavery , “Skin Temperature Monitoring Reduces the Risk for Diabetic Foot Ulceration in High‐Risk Patients,” The American journal of medicine 120, no. 12 (2007): 1042–1046, 10.1016/j.amjmed.2007.06.028. 18060924

[17] J.‐H. Yue , S.‐J. Zhang , Q. Sun , et al., “Local Warming Therapy for Treating Chronic Wounds,” Medicine 97, no. 12 (2018): 9931, 10.1097/MD.0000000000009931. PMC5895350 29561463

[18] Z. Sun , J. Yue , and Q. Zhang , “Local Warming Therapy for Treating Chronic Wounds,” The Cochrane Database of Systematic Reviews 2017 (2017): CD011728.

[19] M. Kamaraj , N. Moghimi , J. Chen , et al., “New Dimensions of Electrospun Nanofiber Material Designs for Biotechnological Uses,” Trends in Biotechnology 42, no. 5 (2024): 631–647, 10.1016/j.tibtech.2023.11.008. 38158307 PMC11065627

[20] Y. Qiu , J. Tian , S. Kong , et al., “SrCuSi 4 O 10 /GelMA Composite Hydrogel‐Mediated Vital Pulp Therapy: Integrating Antibacterial Property and Enhanced Pulp Regeneration Activity,” Advanced Healthcare Materials 12 (2023): 2300546, 10.1002/adhm.202300546. 37260366 PMC11469286

[21] Q. Yu , J. Chang , and C. Wu , “Silicate Bioceramics: from Soft Tissue Regeneration to Tumor Therapy,” Journal of Materials Chemistry B 7, no. 36 (2019): 5449–5460, 10.1039/C9TB01467E. 31482927

[22] Y. Zhou , C. Wu , and J. Chang , “Bioceramics to Regulate Stem Cells and Their Microenvironment for Tissue Regeneration,” Materials Today 24 (2019): 41–56, 10.1016/j.mattod.2018.07.016.

[23] A. Ratep and I. Kashif , “X‐ray Photoelectron, FTIR, and Mössbauer Spectroscopy Studied the Effect of Fe_2_O_3_/CuO Substitution on Structural and Electrical Properties of Lithium Borosilicate Glasses,” Journal of Materials Science: Materials in Electronics 32, no. 9 (2021): 12340–12347.

[24] C. Parvathiraja and S. Shailajha , “Bioproduction of CuO and Ag/CuO Heterogeneous Photocatalysis‐Photocatalytic Dye Degradation and Biological Activities,” Applied Nanoscience 11, no. 4 (2021): 1411–1425, 10.1007/s13204-021-01743-5.

[25] F. El‐Sayed , M. S. A. Hussien , T. H. AlAbdulaal , et al., “Study of Catalytic Activity of G‐SrO Nanoparticles for Degradation of Cationic and Anionic Dye and Comparative Study Photocatalytic and Electro & Photo‐electrocatalytic of Anionic Dye Degradation,” Journal of Materials Research and Technology 20 (2022): 959–975, 10.1016/j.jmrt.2022.07.108.

[26] I. Garofano , A. Duran , J. L. Perez‐Rodriguez , and M. D. Robador , “Natural Earth Pigments from Roman and Arabic Wall Paintings Revealed by Spectroscopic Techniques,” Spectroscopy Letters 44, no. 7‐8 (2011): 560–565, 10.1080/00387010.2011.610655.

[27] J. V. John , N. S. Sharma , G. Tang , et al., “Nanofiber Aerogels with Precision Macrochannels and LL‐37‐Mimic Peptides Synergistically Promote Diabetic Wound Healing,” Advanced functional materials 33, no. 1 (2023): 2206936, 10.1002/adfm.202206936. 36714167 PMC9881731

[28] J. V. John , A. McCarthy , H. Wang , et al., “Freeze‐Casting with 3D‐Printed Templates Creates Anisotropic Microchannels and Patterned Macrochannels within Biomimetic Nanofiber Aerogels for Rapid Cellular Infiltration,” Advanced healthcare materials 10, no. 12 (2021): 2100238, 10.1002/adhm.202100238. PMC8222158 34029004

[29] D. Cibrian , H. de la Fuente , and F. Sánchez‐Madrid , “Metabolic Pathways That Control Skin Homeostasis and Inflammation,” Trends in Molecular Medicine 26, no. 11 (2020): 975–986, 10.1016/j.molmed.2020.04.004. 32371170

[30] X. Dong , J. Ye , Y. Chen , T. Tanziela , H. Jiang , and X. Wang , “Intelligent Peptide‐Nanorods Against Drug‐Resistant Bacterial Infection and Promote Wound Healing by Mild‐Temperature Photothermal Therapy,” Chemical Engineering Journal 432 (2022): 134061, 10.1016/j.cej.2021.134061.

[31] S. H. Beachy and E. A. Repasky , “Toward Establishment of Temperature Thresholds for Immunological Impact of Heat Exposure in Humans,” International Journal of Hyperthermia 27, no. 4 (2011): 344–352, 10.3109/02656736.2011.562873. 21591898 PMC3620730

[32] G. Khachaturyan , A. W. Holle , K. Ende , et al., “Temperature‐Sensitive Migration Dynamics in Neutrophil‐Differentiated HL‐60 Cells,” Scientific Reports 12 (2022): 7053, 10.1038/s41598-022-10858-w. 35488042 PMC9054779

[33] H. Izumi , K. Sato , K. Kojima , T. Saito , T. C. Saido , and K. Fukunaga , “Oral Glutathione Administration Inhibits the Oxidative Stress and the Inflammatory Responses in AppNL−G‐F/NL−G‐F Knock‐in Mice,” Neuropharmacology 168 (2020): 108026, 10.1016/j.neuropharm.2020.108026. 32130977

[34] J. Muri and M. Kopf , “Redox Regulation of Immunometabolism,” Nature Reviews Immunology 21, no. 6 (2021): 363–381, 10.1038/s41577-020-00478-8. 33340021

[35] C. Wang , E. Shirzaei Sani , C.‐D. Shih , et al., “Wound Management Materials and Technologies from Bench to Bedside and Beyond,” Nature Reviews Materials 9, no. 8 (2024): 550–566, 10.1038/s41578-024-00693-y. PMC12176411 40535534

[36] R. Yang , F. Liu , J. Wang , X. Chen , J. Xie , and K. Xiong , “Epidermal Stem Cells in Wound Healing and Their Clinical Applications,” Stem Cell Research & Therapy 10, no. 1 (2019): 229, 10.1186/s13287-019-1312-z. 31358069 PMC6664527

[37] R. Fan , C. Zhang , F. Li , et al., “Hierarchically Assembled Nanofiber Scaffolds with Dual Growth Factor Gradients Promote Skin Wound Healing through Rapid Cell Recruitment,” Advanced Science 11, no. 14 (2024): 2309993, 10.1002/advs.202309993. 38326085 PMC11005683

[38] A. R. Bourgonje , M. Feelisch , K. N. Faber , A. Pasch , G. Dijkstra , and H. van Goor , “Oxidative Stress and Redox‐modulating Therapeutics in Inflammatory Bowel Disease,” Trends in molecular medicine 26, no. 11 (2020): 1034–1046, 10.1016/j.molmed.2020.06.006. 32620502

